# Incidence of and risk factors for severe cardiovascular collapse after endotracheal intubation in the ICU: a multicenter observational study

**DOI:** 10.1186/s13054-015-0975-9

**Published:** 2015-06-18

**Authors:** Sebastien Perbet, Audrey De Jong, Julie Delmas, Emmanuel Futier, Bruno Pereira, Samir Jaber, Jean-Michel Constantin

**Affiliations:** Department of Perioperative Medicine, University Hospital of Clermont-Ferrand, Clermont-Ferrand, France; Anesthesiology and Critical Care Medicine, Department B, Saint Eloi Hospital, University Hospital of Montpellier, INSERM U1046, Montpellier, France; Biostatistics Unit (Department of Clinical Research and Innovation), University Hospital of Clermont-Ferrand, Clermont-Ferrand, France

## Abstract

**Introduction:**

Severe cardiovascular collapse (CVC) is a life-threatening complication after emergency endotracheal intubation (ETI) in the ICU. Many factors may interact with hemodynamic conditions during ETI, but no study to date has focused on factors associated with severe CVC occurrence. This study assessed the incidence of severe CVC after ETI in the ICU and analyzed the factors predictive of severe CVC.

**Methods:**

This was a secondary analysis of a prospective multicenter study of 1,400 consecutive intubations at 42 ICUs. The incidence of severe CVC was assessed in patients who were hemodynamically stable (mean arterial blood pressure >65 mmHg without vasoactive drugs) before intubation, and the factors predictive of severe CVC were determined by multivariate analysis based on patient and procedure characteristics.

**Results:**

Severe CVC occurred following 264 of 885 (29.8 %) intubation procedures. A two-step multivariate analysis showed that independent risk factors for CVC included simple acute physiologic score II regardless of age (odds ratio (OR) 1.02, *p* < 0.001), age 60–75 years (OR 1.96, *p* < 0.002 versus <60 years) and >75 years (OR 2.81, *p* < 0.001 versus <60 years), acute respiratory failure as a reason for intubation (OR 1.51, *p* = 0.04), first intubation in the ICU (OR 1.61, *p* = 0.02), noninvasive ventilation as a preoxygenation method (OR 1.54, *p* = 0.03) and inspired oxygen concentration >70 % after intubation (OR 1.91, *p* = 0.001). Comatose patients who required ETI were less likely to develop CVC during intubation (OR 0.48, *p* = 0.004).

**Conclusions:**

CVC is a frequent complication, especially in old and severely ill patients intubated for acute respiratory failure in the ICU. Specific bundles to prevent CVC may reduce morbidity and mortality related to intubation of these high-risk, critically ill patients.

**Trial registration:**

clinicaltrials.gov NCT01532063; registered 8 February 2012.

## Introduction

Severe cardiovascular collapse (CVC) is one of the most frequent, severe life-threatening complications after emergency endotracheal intubation (ETI) in critically ill patients. CVC after ETI is defined by hemodynamic instability (systolic blood pressure ≤65 mmHg recorded at least once and/or ≤90 mmHg for ≥30 minutes despite vascular loading with 500–1000 mL and/or introduction of vasoactive support) [1–4]. ETI in the ICU is most often an unscheduled procedure to treat severe respiratory failure and/or as part of cardiorespiratory resuscitation. Many factors may influence hemodynamic conditions during ETI, including patient medical history and medications, sepsis status, drugs used to induce anesthesia, reason for intubation, and intrathoracic positive pressure related to mechanical ventilation. Risk factors related to serious life-threatening complications include acute respiratory failure and shock as an indication for ETI [1, 5]. To date, however, no study has specifically analyzed factors associated with severe CVC following ETI in the ICU. Early identification of risk factors may enable the use of methods to reduce patient morbidity, including drug treatment, airway management, and additional assistance during intubation procedures [6].

This secondary analysis of a prospective, multiple center observational study performed in 42 ICUs in France (the FRIDAREA study) [7] assessed the incidence of severe CVC after ETI in the ICU as a primary endpoint, and analyzed risk factors predictive of severe CVC in these critically ill patients and evaluated mortality at 28 days as secondary endpoints.

## Methods

### Study design and population

This was a secondary analysis of patients in the FRIDAREA study database [7]. Briefly, FRIDAREA was a prospective, observational, multicenter study conducted in 42 ICUs to develop a model predictive of difficult intubation (original cohort), and in 18 ICUs to validate the model (validation cohort) [7]. All adult patients consecutively intubated in involved ICUs were included. Exclusion criteria were pregnancy, refusal to participate after information was provided or age <18 years. The primary endpoint was the incidence of severe CVC after ETI in the ICU and secondary endpoints were risk factors predictive of severe CVC in these critically ill patients and evaluated mortality at 28 days.

### Ethics and consent

Because of the observational, noninvasive design of this study, the need for written consent was waived. The local ethics committee, the Comité de Protection des Personnes Sud-Mediterranée III, approved the study design (code UF 8819, register 2011-A001122-39).

### Data collection

Clinical parameters were prospectively assessed before, during, and after intubation procedures, with an independent observer collecting variables during and after intubation. Data assessed before intubation included: age; body mass index; severity score (modified Simplified Acute Physiologic Score (SAPS) II at admission, with age eliminated to avoid colinearity with age in the multivariate analysis, as described previously [8, 9]); Sequential Organ Failure Assessment (SOFA) score on the day of the procedure; type of admission (medical versus surgical); co-morbidities such as alcoholism, smoking, cirrhosis, and chronic obstructive pulmonary disease (COPD); cause of admission; cause of intubation (coma was defined as a Glasgow score <8); date and hour of intubation (daytime procedures were those performed from 8 am to 7 pm, with all others defined as on-call procedures); intubation during the previous 2 weeks; nature and number of operators; fluid loading; vasopressor use; noninvasive ventilation (NIV); and emergency characteristics of the intubation (with a real emergency defined as the requirement for immediate ETI, relative emergency as ETI required within 1 hour, and deferred emergency as ETI required in >1 hour). Just before intubation preoxygenation and method of preoxygenation (standard versus NIV) were recorded.

Minimal and maximal heart rate, arterial pressure and saturation were measured before intubation, during intubation (between induction of anesthesia and tube insertion) and within 30 minutes after intubation. Drugs used for intubation were recorded. The MACOCHA score, a seven-item (Mallampati score III or IV, obstructive sleep apnea syndrome, reduced mobility of cervical spine, limited mouth opening, severe hypoxia, coma, nonanesthesiologist as operator) predictor of difficult intubation with a cut-off of 3 points [7], was assessed.

Finally, the percentages of patients undergoing capnography (end-tidal CO_2_ curve) and esophageal intubation; the rates of agitation, aspiration, cardiac arrest, and arrhythmias; ventilation parameters; and 28-day mortality rate were evaluated.

### Definition of severe CVC

Arterial blood pressure was monitored continuously in patients carrying an intra-arterial catheter for 5 of 30 minutes after intubation in patients with cuff measurements. Severe CVC was defined as systolic blood pressure ≤65 mmHg recorded at least once and/or ≤90 mmHg lasting ≥30 minutes despite vascular loading with 500–1000 mL crystalloid and/or colloid solutions and/or a requirement for vasoactive support [1–4]. To avoid confounding factors of hemodynamic variability, only patients who were hemodynamically stable before intubation (defined as mean arterial blood pressure >65 mmHg without vasoactive drugs [10, 11]) were included. Therefore, patients intubated for cardiac arrest and shock were secondarily excluded.

### Statistical analysis

All statistical analyses were performed using Stata software, version 12 (StataCorp, College Station, Tx, USA). The tests were two-sided, with a type I error set at α = 0.05. Mean and standard deviation (SD) or median and interquartile range were calculated for continuous variables, and number of patients and associated percentages were calculated for categorical parameters. Categorical variables were compared between independent groups using the Chi-squared test or Fisher’s exact test, and continuous variables were compared using Student's t-test or the Mann-Whitney test, with normality verified by the Shapiro-Wilk test and homoscedasticity by the Fisher-Snedecor test. Factors significant in the univariate analysis (*p* < 0.1 [12, 13]) and parameters deemed clinically relevant [6, 14, 15], such as use of ketamine or etomidate, COPD and fluid challenge >500 mL (adjustment factors), were included in backward and forward stepwise multivariate logistic regression analyses to determine risk factors independently associated with CVC. The interactions between possible predictive factors were also tested. Results were expressed as odds ratios (ORs) and 95 % confidence intervals (CIs). Multivariate analysis consisted of three steps: 1) patient characteristics (first model), 2) intubation procedures in the ICU (second model), and 3) parameters statistically significant in these two models (model). Univariate analyses identified a cut-off of inspired oxygen concentration (FiO_2_) of 70 % and age in three stages (<60, 60–75 and >75 years) for testing in the multivariate models. The Hosmer-Lemeshow test was used to assess the goodness of fit of the logistic model. A cross-validation process was considered to assess the goodness of fit of the final models obtained. Following these multivariate analyses, a receiver-operating characteristic (ROC) curve associated with the occurrence of CVC (model) was plotted for each proposed model. The statistical power of the final was tested according to works proposed by Tosteson and Demidenko [16, 17].

## Results

**Fig. 1:**
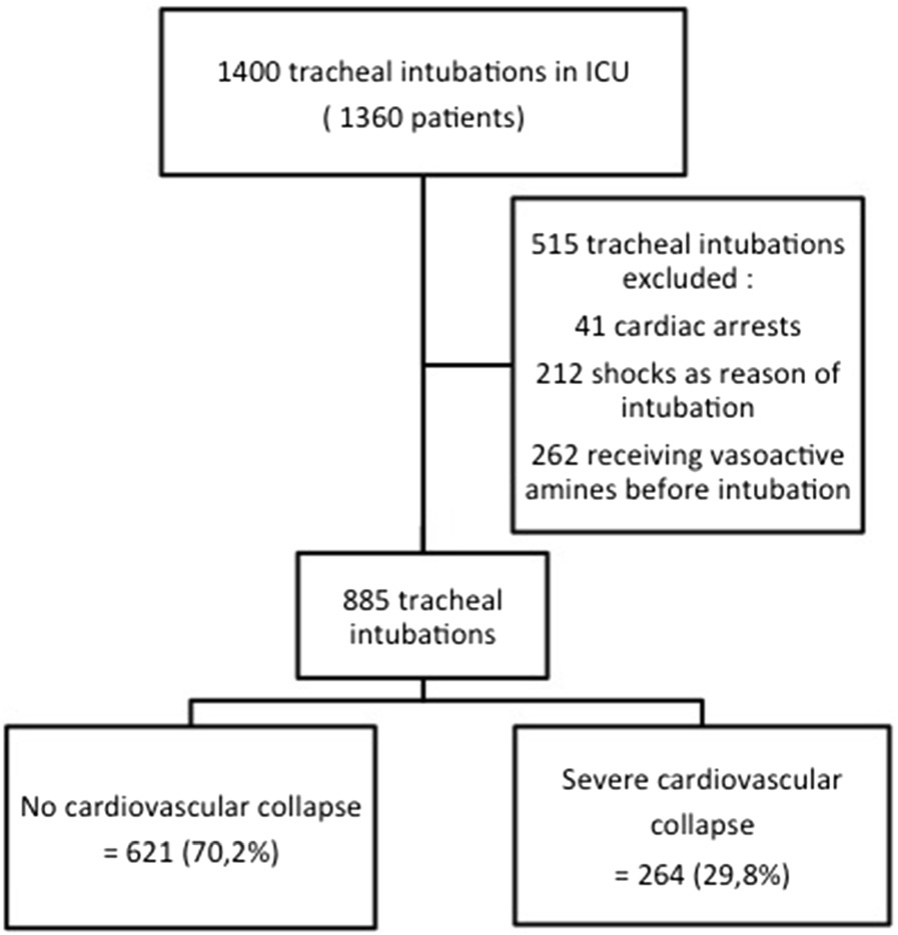
Study flow chart

During the study period, 1,400 intubation procedures were performed in 1,360 patients. After excluding 41 patients who underwent intubation for cardiac arrest, 212 who underwent intubation for shock and 262 who received vasoactive drugs before intubation, 885 intubation procedures were included (Fig. 1).

Severe CVC was observed following 264 of 885 (29.8 %) intubation procedures. The relationships between patient characteristics and the incidence of severe CVC are shown in Table 1. Data on the intubation procedures are detailed in Table 2. Of the 885 intubations, 597 (67 %) were performed due to acute respiratory failure, 241 (27 %) due to coma and 148 (17 %) due to failure of planned extubation. Reasons for the 94 (11 %) other intubations included need for surgical procedures (n = 12, 1.4 %), self-extubation (n = 12, 1.4 %), endoscopy for digestive hemorrhage (n = 29, 3.3 %), uncontrolled agitation (n = 12, 1.4 %), air leak in the balloon probe (n = 4, 0.5 %) and others (n = 25, 2.8 %).

**Table 1:** Characteristics of patients

	Total	No collapse	Collapse	*p*-value
	(n = 885)	(n = 621)	(n = 264)	
Age in years, mean ± SD	58.4 ± 0.6	56.0 ± 0.7	64.0 ± 0.9	<0.0001
Male gender, n (%)	563 (64.6)	387 (63.4)	176 (67.4)	0.28
SAPS II score regardless of age, mean ± SD	36.8 (16.7)	35.8 (16.5)	39.3 (17.1)	0.006
Surgical admission, n (%)	271 (30.6)	195 (31.4)	76 (28.8)	0.47
SOFA score, mean ± SD	5.0 ± 0.1	4.8 ± 0.1	5.3 ± 0.2	0.02
Reason for ICU admission:				
Acute respiratory failure, n (%)	422 (48.7)	260 (41.9)	162 (61.4)	<0.0001
Postoperative complications, n (%)	92 (10.4)	60 (9.7)	32 (12.1)	0.28
Brain injury, n (%)	263 (29.7)	216 (34.8)	47 (17.8)	<0.0001
Acute kidney injury, n (%)	54 (6.1)	40 (6.4)	14 (5.3)	0.65
Trauma, n (%)	62 (7.0)	51 (8.2)	11 (4.2)	0.03
Past medical history				
Tobacco, n (%)	297 (33.6)	205 (33.0)	92 (34.9)	0.64
Cirrhosis, n (%)	83 (9.4)	64 (10.3)	19 (7.2)	0.17
COPD, n (%)	153 (17.3)	95 (15.3)	58 (22.0)	0.02
Obesity, n (%)	163 (18.4)	114 (18.4)	49 (18.6)	0.94
Diabetes, n (%)	131 (14.8)	89 (14.3)	42 (15.9)	0.55
Reasons for intubation				
Coma, n (%)	241 (27.2)	194 (31.2)	47 (17.8)	<0.0001
Acute respiratory failure, n (%)	597 (67.5)	375 (60.4)	222 (84.1)	<0.0001
Extubation failure, n (%)	148 (16.7)	111 (17.9)	37 (14.0)	0.17
Other, n (%)	94 (10.6)	83 (13.4)	11 (4.2)	0.88

*COPD* chronic obstructive pulmonary disease, *SAPS* Simplified Acute Physiologic Score, *SD* standard deviation, *SOFA* Sequential Organ Failure Assessment

**Table 2:** Characteristics of intubation procedures

	Total	No collapse	Collapse	*p*-value
	(n = 885)	(n = 621)	(n = 264)	
First intubation, n (%)	543 (61.4)	364 (58.6)	179 (67.8)	0.01
Anesthesiologist, n (%)	580 (65.5)	415 (66.8)	165 (62.5)	0.22
Fluid challenge, n (%)	336 (38.0)	229 (36.9)	107 (40.5)	0.33
SpO_2_ before ETI, mean ± SD	87.72 ± 0.5	88.4 ± 0.6	86.13 ± 0.8	0.03
NIV (out of preoxygenation), n (%)	324 (36.6)	185 (29.8)	139 (52.7)	<0.0001
Drug for induction, n (%)	863 (97.5)	605 (97.4)	258 (97.7)	0.79
Nesdonal, n (%)	25 (2.8)	17 (2.7)	8 (3.0)	0.81
Propofol, n (%)	139 (15.7)	111 (17.9)	28 (10.6)	0.01
Dose mg/kg, mean ± SD	2.13 ± 1.23	2.10 ± 1.23	2.27 ± 1.27	0.54
Etomidate, n (%)	421 (47.6)	286 (46.1)	135 (51.1)	0.17
Dose mg/kg, mean ± SD	0.45 ± .44	0.47 ± 0.46	0.42 ± 0.41	0.37
Ketamine, n (%)	188 (21.2)	124 (20.0)	64 (24.2)	0.16
Dose mg/kg, mean ± SD	2.77 ± 1.06	2.82 ± 1.10	2.66 ± 0.98	0.31
Other, n (%)	39 (4.4)	24 (3.9)	15 (5.7)	0.23
Opioids, n (%)	74 (8.4)	53 (8.5)	21 (8.0)	0.78
Fentanyl, n (%)	8 (0.9)	5 (0.8)	3 (1.1)	0.63
Sufentanil, n (%)	53 (6.0)	42 (6.8)	11 (4.2)	0.14
Remifentanil, n (%)	9 (1.0)	6 (1.0)	3 (1.1)	0.82
Other, n (%)	3 (0.3)	3 (0.48)	0 (0)	0.26
NMBA, n (%)	770 (87.0)	543 (87.4)	227 (86.0)	0.56
Suxamethonium, n (%)	646 (73.0)	446 (71.8)	200 (75.8)	0.23
Rocuronium, n (%)	92 (10.4)	69 (11.1)	23 (8.7)	0.29
Other, n (%)	45 (5.1)	32 (5.2)	13 (4.9)	0.89
MACOCHA score, n (%)				0.37
<3	544 (83.9)	385 (84.8)	159 (82.0)	
≥3	104 (16.1)	69 (15.2)	35 (18.0)	
Preoxygenation, n (%)	841 (95.0)	585 (94.2)	256 (97.0)	0.08
Duration of preoxygenation, mean ± SD	1.23 (0.7)	1.21 (0.7)	1.29 (0.7)	0.11
NIV for preoxygenation, n (%)	371 (41.9)	233 (37.5)	138 (52.3)	< 0.0001
Incident during ETI, n (%)				
Inhalation, n (%)	102 (11.5)	66 (10.6)	36 (13.6)	0.20
Cardiac rhythm abnormalities, n (%)	15 (1.7)	8 (1.3)	7 (2.7)	0.15
Desaturation, n (%)	177 (20.0)	101 (16.3)	76 (28.8)	< 0.0001
Implementation of sedation, n (%)	802 (90.6)	554 (89.2)	248 (93.9)	0.03
FIO_2_, mean ± SD	68.52 ± 0.9	65.57 ± 1.1	75.11 ± 1.5	< 0.0001
Tidal volume, mean ± SD	458.5 ± 2.9	459.01 ± 3.4	457.35 ± 5.1	0.79
PEEP, mean ± SD	5.76 ± 0.1	5.65 ± 0.1	6.01 ± 0.1	0.02
RM, n (%)	108 (12.2)	68 (11.0)	40 (15.2)	0.08

*FiO*_*2*_ inspired oxygen concentration, *MACOCHA score* a seven-item (Mallampati score III or IV, obstructive sleep apnea syndrome, reduced mobility of cervical spine, limited mouth opening, coma, severe hypoxia, nonanesthesiologist as operator) simplified score, *NMBA* neuromuscular blocking agents, *PEEP* positive end-expiratory pressure, *RM* recruitment maneuver, *SD* standard deviation, *SpO*_*2*_ oxygen saturation, *NIV* noninvasive ventilation

Univariate analysis showed that risk factors for CVC included patient age, SAPS II score regardless of age, SOFA score, COPD, acute respiratory failure as a reason for ICU admission and intubation, initial intubation in the ICU, hypoxemia before intubation, NIV before intubation (for ventilator support and/or only for preoxygenation), desaturation during the intubation procedure, FiO_2_ >70 % after intubation, administration of sedation immediately after intubation and ventilation with positive end-expiratory pressure (PEEP) of 6 cmH_2_O. Brain injury as a reason for ICU admission, coma as a reason for intubation and propofol (whatever the dosage per kg) to induce anesthesia were identified as protective factors of CVC. The 28-day mortality rate was significantly higher in patients who did than did not experience CVC (30.4 % versus 19.3 %, *p* = 0.001).

In the first multivariate model, which included patient characteristics, SAPS II regardless of age (OR 1.02, 95 % CI 1.01–1.03, *p* = 0.005), age 60–75 years (OR 2.07, 95 % CI 1.39–3.10, *p* < 0.001 versus <60 years) and >75 years (OR 2.77, 95 % CI 1.73–4.43, *p* < 0.001 versus <60 years), acute respiratory failure as a reason for intubation (OR 1.56, 95 % CI 1.05–2.30, *p* = 0.03) and initial intubation in the ICU (OR 1.64, 95 % CI 1.12–2.40, *p* = 0.01) were independent risk factors for CVC, whereas coma as a reason for intubation was a protective factor (OR 0.51, 95 % CI 0.31–0.86, *p* = 0.01). A history of COPD was not significantly associated with CVC occurrence (OR 1.26, 95 % CI 0.80–1.97, *p* = 0.31). Brain injury as a reason for ICU admission was not an independent protective factor (OR 0.72, 95 % CI 0.43–1.19, *p* = 0.20).

In the second model, which included parameters associated with intubation procedures, independent risk factors for severe CVC occurrence were NIV as a preoxygenation method (OR 1.80, 95 % CI 1.24–2.59, *p* = 0.002) and FiO_2_ >70 % after intubation (OR 1.84, 95 % CI 1.29–2.61, *p* = 0.002). Use of ketamine (OR 1.61, 95 % CI 0.86–2.99, *p* = 0.14), use of etomidate (OR 1.35, 95 % CI 0.78–2.32, *p* = 0.29), use of propofol (OR 0.69, 95 % CI 0.35–1.37, *p* = 0.29), administration of sedation immediately after intubation (OR 1.27, 95 % CI 0.67–2.41, *p* = 0.47), and fluid challenge >500 mL (OR 1.20, 95 % CI 0.78–1.85, *p* = 0.42) were not significantly associated with CVC occurrence.

**Fig. 2:**
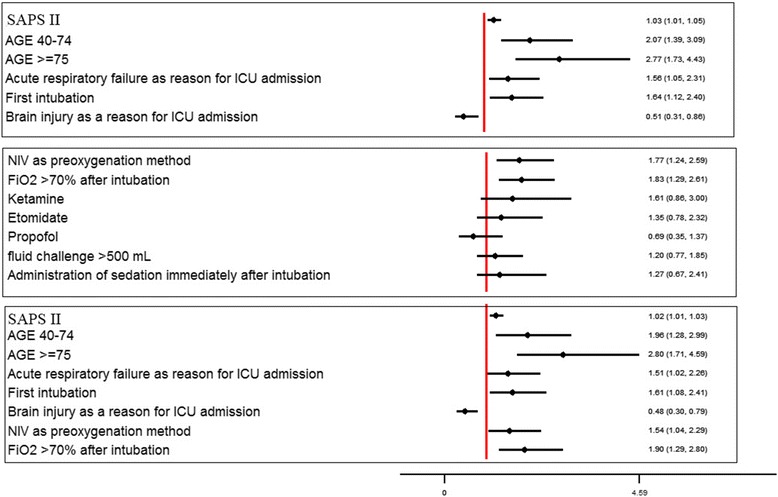
Forrest plot. *FiO2* inspired oxygen concentration, *NIV* noninvasive ventilation, *SAPS* Simplified Acute Physiologic Score

The third multivariate analysis showed that independent risk factors for CVC included SAPS II score regardless of age (OR 1.02, 95 % CI 1.01–1.03, *p* < 0.001), age 60–75 years (OR 1.96, 95 % CI 1.28–2.99, *p* < 0.002 versus <60 years) and >75 years (OR 2.81, 95 % CI 1.72–4.59, *p* < 0.001 versus <60 years), acute respiratory failure as a reason for intubation (OR 1.51, 95 % CI 1.01–2.26, *p* = 0.04), initial intubation in the ICU (OR 1.61, 95 % CI 1.08–2.41, *p* = 0.02), NIV as preoxygenation method (OR 1.54, 95 % CI 1.04–2.29, *p* = 0.03) and FiO_2_ >70 % after intubation (OR 1.91, 95 % CI 1.30–2.80, *p* = 0.001). Coma as a reason for intubation was independently associated with protection against CVC (OR 0.48, 95 % CI 0.30–0.79, *p* = 0.004) (Fig. 2).

### ROC curves and statistical power

Analysis of ROC curves associated with CVC for each model showed that the area under the curve was more important for the third model (0.71) than for the first (0.68) and second (0.64) models. The statistical power of the final model was greater than 85 %. Except for the parameter “first intubation”, the statistical power was greater than 90 % for each predictive factor presented in the final model.

## Discussion

This large cohort analysis showed that CVC was a frequent complication of intubation in the ICU. Patient age, SAPS II score regardless of age, intubation for acute respiratory failure, initial intubation in the ICU and FiO_2_ >70 % after intubation, but not COPD, were found to be independent risk factors for CVC. Moreover, CVC was associated with a significantly higher 28-day mortality rate.

The CVC rate observed in our study cohort was similar to those reported previously [1], especially in patients without preexisting hypotension before intubation (29 % of 84 patients) [3], or if 15 % were receiving vasopressor before intubation (33 % of 794 patients) [18]. The CVC rate may be dependent on the definition of CVC, particularly the level and duration of hypotension. Based on previous studies, we arbitrarily defined CVC as an arterial systolic blood pressure ≤65 mmHg recorded at least once and/or ≤90 mmHg lasting ≥30 minutes despite vascular loading with 500–1000 mL of crystalloid and/or colloid solutions and/or necessitating introduction of vasoactive drugs [1, 18, 19]. Mortality in the ICU was not related to hypotension after intubation, regardless of its definition and severity [18].

The three models used in our multivariate analyses were built to propose an approach to risk factors for CVC [12]. The first model included patient characteristics, the second model included details of intubation procedures, and the third model included both sets of factors.

Elevated SAPS II score, a good surrogate for illness severity and well correlated with patient mortality, was found to be a risk factor for CVC [20]. Early intubation within the first 24 hours of ICU admission was associated with a high SAPS II score. We did not include age as a component of the SAPS II score to avoid colinearity in the multivariate analyses [8, 9]. A first intubation in ICU was another risk factor. It could follow an uncontrolled evolution of the reason of ICU admission (acute respiratory failure, for example), possibly after NIV failure. In contrast, subsequent intubations may follow extubation failure following the correct treatment of initial shock and multiorgan failure [21].

Acute respiratory failure has been identified as a risk factor for CVC and for complications related to intubation [1, 3, 22, 23]. Desaturation time during apnea associated with intubation may be reduced in ICU patients, especially in hypoxemic patients [24, 25]. Patients with acute respiratory failure have limitations in oxygen transport, alveolar volume and enhanced shunt fraction. Hemoglobin desaturation has been found to increase mortality rates in this population [26, 27]. Preoxygenation with NIV and elevated postintubation FiO_2_ (>70 %) reflect the severity of respiratory failure.

Although fluid challenge before intubation was not significantly associated with CVC in univariate analysis, it was included in multivariate analysis. Its inclusion was justified by the results of a before/after study comparing the implementation of different treatments and procedures during the intubation procedure [6]. Fluid challenge may be a marker of preload dependence or hemodynamic status and may correspond to a prior severe hemodynamic condition characterized by a potential hypovolemic status before induction. These results are complicated by differences in fluid challenge among the ICUs surveyed. Fluid challenge before intubation is systematic in some ICUs, according to the aforementioned bundle, but is administered only to patients with hypovolemia in other ICUs.

Etomidate and ketamine are anesthetic drugs that have a rapid onset and short half-life, are well tolerated hemodynamically and improve intubation conditions [28, 29]. Increased induction with etomidate or ketamine in the ICU from 35 % to 76 % was associated with a significant reduction in the incidence of severe hypotension [6]. In this observational study, etomidate and ketamine were associated with CVC in univariate analysis but not in multivariate analysis. The lack of correlation between the incidence of CVC and administration of these drugs suggests that etomidate and ketamine were chosen for the most severely ill patients because of their hemodynamic safety profiles [6, 30–32].

A previous study showed that implementation of an intubation management protocol reduced the incidence of intubation-related ICU complications, in particular CVC (15 % versus 27 %) [6]. This protocol included fluid challenge before intubation, preoxygenation with NIV, rapid sequence induction (with ketamine or etomidate, and suxamethonium) and early administration of sedation and vasopressors if needed. Our univariate analysis showed that early administration of sedation was significantly associated with CVC. Its nonsignificance on multivariate analysis suggests that it was probably a confounding factor due to patient severity.

Comatose patients who required ETI were less likely to develop CVC during intubation. Indeed, most comatose patients experience failure of only one organ [33]. Furthermore, laryngoscopy and intubation after rapid sequence induction in these patients often results in hypertension, as most patients intubated after rapid sequence induction show a sympathetic response to laryngeal stimulation, characterized by tachycardia and increases in mean arterial pressure and intracranial pressure [33, 34].

The study had several limitations. First, it was not designed to identify factors protective against CVC. Indeed, patients with hemodynamic instability before ETI were not included in this analysis. The addition of such patients may modify the interpretation of these analyses; however, the rate of life-threatening complications after ETI in patients with septic shock before ETI (about 36 %) was similar to the rate reported in nonselected critically ill patients [35]. These patients must be evaluated in future studies. Several of the factors found to be significant in univariate analysis were not included in the multivariate models, for statistical or clinical reasons. For example, PEEP level was not clinically relevant (5.7 versus 6.0 cmH_2_O). Another limitation was our inability to evaluate the correlation between the doses of drugs used for ETI (ketamine, etomidate, thiopental and/or propofol) with the degree of hypotension. The specific association between drugs used to facilitate intubation and severe CVC requires further study. COPD and hypercarbic status have been regarded as independently associated with life-threatening hypotension after intubation, with elevated CO_2_ levels causing generalized vasodilatation [3, 36]. Another limitation is that the presence of an arterial catheter for invasive blood pressure measurement was not fulfilled. It could be a prerequisite and a very important safety measure [6]. Hypercarbia causes sympathetic stimulation, increasing cardiac output secondary to tachycardia [36]. Unfortunately, this study was not designed to record levels of CO_2_ before and after intubation; only the presence of end-tidal CO_2_ curve was noted. Thus, we could not determine the role of CO_2_ variations on CVC occurrence.

## Conclusion

This is the first study designed to specifically analyze independent risk factors for severe CVC after ETI in the ICU. Physicians must be aware that tracheal intubation of an old and severe patient for acute respiratory failure leaves them at high risk for severe CVC. Use of specific bundles to prevent CVC may decrease morbidity and mortality related to intubation of these critically ill patients.

## Key messages

- This study is the first to specifically report independent risk factors for severe cardiovascular collapse (CVC) after endotracheal intubation (ETI) in the intensive care unit. ETI of old and critically ill patients for acute respiratory failure carries a high risk of severe CVC. Use of specific bundles to prevent CVC may decrease morbidity and mortality related to intubation of these critically ill patients.

## Abbreviations

CI: Confidence interval
COPD: Chronic obstructive pulmonary disease
CVC: Cardiovascular collapse
ETI: Endotracheal intubation
FiO_2_: Inspired oxygen concentration
NIV: Noninvasive ventilation
OR: Odds ratio
PEEP: Positive end-expiratory pressure
ROC: Receiver operating characteristic
SAPS: Simplified Acute Physiologic Score
SD: Standard deviation
SOFA: Sequential Organ Failure Assessment
